# Bite mechanics within the wolf lineage: feeding capabilities of *Canis borjgali* and *Canis mosbachensis* inferred by digital bite simulation

**DOI:** 10.1098/rsos.261396

**Published:** 2026-10-07

**Authors:** Emanuele Peri, Alberto Corridori, Saverio Bartolini-Lucenti, David Lordkipanidze, Lorenzo Rook

**Affiliations:** ^1^ Earth Sciences Department, Paleo[Fab]Lab, University of Florence, Florence, Tuscany 50121, Italy; ^2^ Georgian National Museum, Tbilis 0105, Georgia; ^3^ Department of Natural History, Tbilisi State University, Tbilisi 0179, Georgia

**Keywords:** carnivora, canidae, early pleistocene, feeding capabilities, bite biomechanics, finite element analysis (FEA)

## Abstract

Genus *Canis* species plays a key role as predator in terrestrial ecosystems. However, the evolutionary history of these carnivorans is still not fully understood. To explore this topic, we studied the bite of two fossil close relatives of the modern grey wolf, *Canis borjgali* and *Canis mosbachensis*, through digital bite simulations via finite element analysis (FEA). We used computer simulations based on computed tomography (CT) scans of fossil skulls to recreate how these animals catch and chew their prey. These simulations allowed us to test how their skulls react during different types of bites. To improve our analysis, the bite performance of *C. borjgali* and *C. mosbachensis* was compared with those of a selection of living canids having different diets. Our results suggest that *C. borjgali* had a relatively strong skull capable of handling hard food (such as bones). On the other hand, *C. mosbachensis* showed higher stress on its skull during the bite simulations, which means that its skull was somewhat weaker. Moreover, the FEA bite simulation evidence shows that the frontal sinuses (a pair of cavities located in the frontal bones) play a key role in dealing with bite-related stress, especially in a canine bite scenario.

## 1. Introduction

The family Canidae is widely known, as it includes species with a broad distribution and is of interest for human activities. Nowadays, this family plays an important role in modern environments owing to its high adaptability to many ecologies. Indeed, it includes hypocarnivorous (less than 50% of the diet consists of vertebrate meat), mesocarnivorous (the diet consists of 50–70% vertebrate meat) and hypercarnivorous (more than 70% of the diet is of vertebrate meat) predators living all around the world [1]. Furthermore, this family of carnivores now inhabits almost all the continents, and its members spread in a short time span (5.5–0.5 Ma) [2]. Canidae originated in North America in the late Priabonian (Late Eocene) [3,4], and by the end of the Oligocene they were differentiated into three subfamilies: Hesperocyoninae, Borophaginae and Caninae [5,6]. The extinct Hesperocyoninae and Borophaginae roamed exclusively in North America, while Caninae spread to the rest of the world after the emergence of, respectively, the Bering Strait in the Late Miocene and the Isthmus of Panama in the Late Pliocene [6,7,8]. Given the ecological relevance of the family Canidae, the investigation of their complex and partly unresolved evolutionary history appears as a mandatory task for the reconstruction of past environments.

Extant Caninae are subdivided into the clades Vulpini and Canini; the latter can be further separated into the tribes Canina (worldwide distributed) and Cerdocyonina (South American canids) [6]. Canina is one of the most widespread groups and, within this tribe, the genus *Canis* certainly represents one of the most successful. Canini appeared between 6 and 5.5 Ma in North America with jackal-sized forms, such as *Eucyon ferox* and *Canis lepophagus* [9]. Canini rapidly dispersed into the Old World during the Late Miocene (the ‘*Eucyon* event’) with occurrences like *Eucyon davisi* in Asia, *Eucyon monticinensis* in Europe and *Eucyon intrepidus* in Africa [2]. According to the historical interpretation, the genus *Canis* colonized Western Europe during the so-called ‘Wolf event’ (2.0–1.8 Ma) [10,11], but further studies suggest a diachrony of this event and an earlier appearance of this genus [2,12]. Among the earliest better-characterized taxa that reached Europe in the latest Late Pliocene and the Early Pleistocene, there are *Canis etruscus* (which was found in many European sites) [13,14] and *Xenocyon falconeri* [15,16]. After the Wolf event, different forms emerged in Europe and Africa, such as *Canis arnensis* (2.2–1.7 Ma) and *Xenocyon africanus* (approx. 1.7 Ma) [2,16–18]. The traditional interpretation sees *Canis lupus* (the grey wolf) as the extant species of the evolutionary line that started with *C. etruscus* and was followed by *Canis mosbachensis* (Early to Middle Pleistocene) [14,19]. However, this paradigm has been recently questioned by the description of *Canis borjgali*, a wolf-like canid similar in size to *C. etruscus* from the Early Pleistocene of Dmanisi (Georgia, 1.85–1.76 Ma) [14]. In such a changing and intricate context, the investigation of the link between cranial structure and feeding capabilities appears as a fundamental tool to resolve the evolutive dynamics of the wolf lineage.

Following this direction, in this study we simulated the bite of *C. borjgali* and *C. mosbachensis*, two close relatives of the extant grey wolf, to collect new clues about their feeding capabilities. These two species are chronostratigraphically close but geographically distant, and these conditions make the investigation of their feeding adaptations particularly intriguing and useful for understanding the evolution of the wolf lineage. Here, finite element analysis (FEA) was used to simulate the bite of these fossil species (figure 1). Such a digital technique allows the estimation of the mechanical behaviour of a body under a load, and it is a widely used method for the exploration of the link between form and function both in extant and fossil Carnivora [20–26,27].

**Figure 1.**
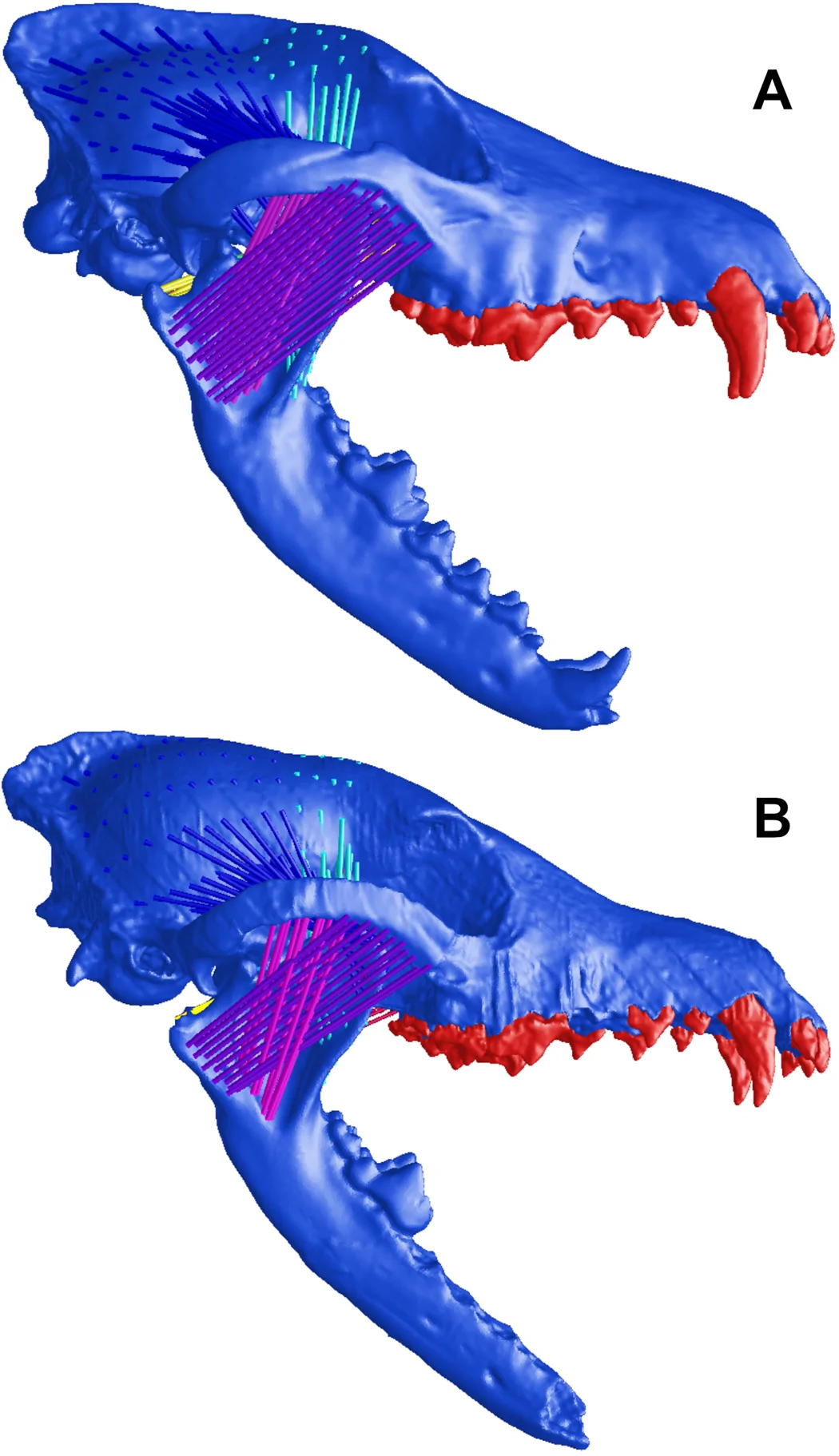
Solid meshes of (A) *C. borjgali* and (B) *C. mosbachensis* used for the bite simulations with the pretensioned beam elements representing the mandible elevator muscles (light blue for the pars superficialis of the temporalis, dark blue for the pars profunda of the temporalis, dark purple for the pars superficialis of the masseter, light purple for the pars profunda of the masseter, yellow is for the medial pterygoid). The blue bricks are for the bone; the red bricks are for the dentine.

The mechanical behaviour of the crania in *C. borjgali* and *C. mosbachensis* was compared with a broad sample of living Canidae to find common features in the stress patterns and to infer their feeding habits. In addition, this study aims to assess the influence of the frontal sinuses on cranial mechanics during a biting action in Canidae. These are a pair of cavities included in the paranasal sinuses, grossly located between the postorbital processes. The frontal sinuses are present in many Carnivora (e.g. Ursidae, Felidae and several Canidae) with varying degrees of development, but their function is still debated [28–31,32]. Among the most widely accepted hypotheses, there is the dissipation of the stress on the cranium generated by a biting action [28,29,32]. Interestingly, recent quantitative and biomechanical studies focused on the relationship between frontal sinuses and bite mechanics in canids support this view [27,32]. The results of this study suggest that *C. borjgali* displays a stiffer cranium than *C. mosbachensis*, and that this species was more adapted to process hardened food items. Furthermore, the simulations presented here confirm the role played by the frontal sinuses in the bite mechanics. These new data expand our knowledge on the feeding capabilities of two important ancestors of *C. lupus* and help to reconstruct its ecological evolution.

## 2. Results

The von Mises stress patterns resulting from the bite simulations are shown in figure 2. In all the load cases, two evident stress peaks are displayed on the zygomatic arch, ranging from 19.5 MPa to more than 30 MPa in some species. One peak is placed at the base of the zygomatic process of the temporal, and one is located at its mid-length.

**Figure 2.**
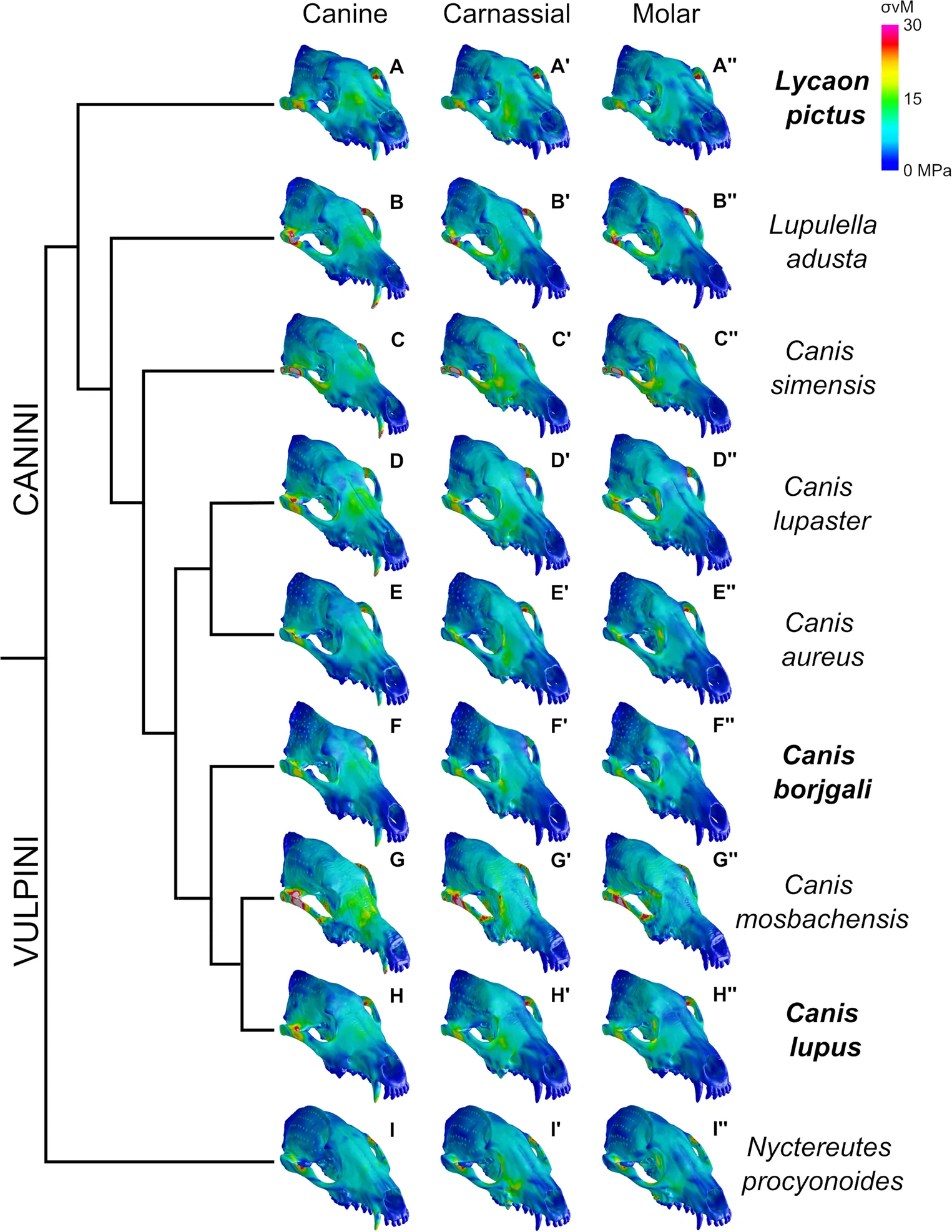
Distribution map of von Mises stress (σvM) of *C. borjgali*, *C. mosbachensis* and extant canids. (A–I) bite simulations at the canine teeth, (A′–I′) bite simulations at the carnassial tooth and (A″–I″) bite simulations at the M1 tooth. The crania are in anterolateral view and not to scale. Note that the dotted pattern on the neurocranium is due to the punctiform force exerted by the trusses simulating the muscle fibres. The forms with the most developed frontal sinuses are shown in bold. The species are arranged according to simplified phylogenetic relationships following Zrzavý *et al.* [33]. The relationship between *C. borjgali, C. mosbachensis* and *C. lupus* are personal observations from the authors.

*Canis borjgali* in the simulations at the canines (figure 2F) exhibits a broad area with low stress values (from 5.5 to 6.5 MPa) and a uniform aspect affecting the cranium between the antorbital foramina. Such an area extends anteriorly at the level of the second premolar and posteriorly at the rostralmost position of the frontals. There, three more stressed spots can be detected (green in figure 2F, values between 10.5 and 13 MPa): one grossly placed at the caudal end of the nasals and two symmetrical at the orbital margins. Moving toward the caudal end of the cranium, there is an evident fall in the von Mises stress values on the dorsal surface of the frontals between the postorbital processes, and the stress pattern takes on a heterogeneous appearance. This low-stress portion corresponds to the frontal sinuses, and the morphology of their bony struts traces well the pattern of the lowest stress values. On the neurocranium, there is a wide stressed area with relatively low values (from 5.5 to 6.5 MPa) affecting the ventral portions of the frontal and the temporal, as well as the lateral wall of the orbit. As a result of the bending action on the snout, even the anteriormost portion of the sagittal crest displays low stress values. In the results of the carnassial bite simulation of *C*. *borjgali* (figure 2F′), the most evident feature is a wide stressed area that extends from the right third premolar to the posterior end of the right frontal, exhibiting von Mises stress values from low (5–8 MPa) to moderate (11–17.5 MPa) at the level of the carnassial tooth. Such a stressed area mainly affects the right side of the cranium, where the dental constraint was placed, making the von Mises stress pattern asymmetrical. Even the margin of the right orbit is moderately stressed, probably acting as a counterbalance against the displacement. The left side of the cranium is far less affected at the base of the snout and on the frontal, with generally lower values than the right side and just a minor peak on the anterior margin of the orbit. On the neurocranium, a somewhat opposite situation can be observed, with the left side more stressed than the right, as a reaction to the presence of a single constraint on the right carnassial tooth. There is a clear stress peak (12.5–14 MPa) on the temporal in correspondence with the eminence of the zygomatic process. In addition to this, there is a wide low-stress region that includes the ventral portion of the temporal and frontal. On both sides of the cranium, the lateral walls of the orbits exhibit relatively low stress values. The von Mises stress pattern resulting from the bite simulation at the first molar (figure 2F″) is roughly like that observed at the carnassial tooth. However, the peak observed above the carnassial tooth is less intense (11–13.5 MPa). Moreover, the stress at the margin on the right orbit is mainly limited to its anteroventral portion, with peak values close to 20 MPa. Another feature is a moderately stressed area with values between 11 and 15.5 MPa affecting the lateral wall of the orbit. According to the diagrams describing the stress trends on the dorsal surface of the crania (figure 3), *C. borjgali* generally shows low values compared with those of the other species included here. Moreover, the line describing the trend on the dorsal surface of this fossil form does not show the peaks observable in all the other canids considered here. These data, along with the pattern of the von Mises stress distribution, suggest for *C. borjgali* a cranial structure refractory to the bite-related loads. Comparing the distribution of the von Mises stress across the three load cases in the *C. borjgali* specimen tested here with those observed in the extant sample, the greatest similarities emerge with *C. lupus* (figure 2H–H″), both for shape of the patterns and values. Indeed, *C. borjgali* displays stress peaks remarkably close (both for position and values) to those observed on *C. lupus* at the base of the snout in the canine bite simulation and on the right maxilla in the carnassial bite simulation (figure 2F,F′,H,H′). Moreover, the shape of the stress patterns on the snout and on the frontal region in *C*. *borjgali* strongly recalls the results obtained for *C*. *lupus*. However, in the molar bite simulation, the stress peaks observed on MG-GNM-D64 at the level of the orbital margin and lateral wall (figure 2F″) are less intense compared with the *C*. *lupus* specimen examined here (figure 2H″). In addition, the stress patterns on the neurocranium of *C*. *borjgali* are less extensive than those resulting on the *C*. *lupus* considered in this study. Based on these data, the cranium of *C*. *borjgali* appears slightly stiffer than that of *C*. *lupus*, despite their great similarities in mechanical behaviour. Minor similarities can be detected even in the stress patterns of *C*. *aureus*. Indeed, in the bite simulations of the golden jackal, we found stress peaks that resemble those on *C*. *borjgali* for the position, but less so for intensity. Furthermore, in the canine bite simulations of *C. aureus* and *C. borjgali*, there is a similar fall in the stress values on the dorsal surface of the frontals in correspondence with the frontal sinuses.

**Figure 3.**
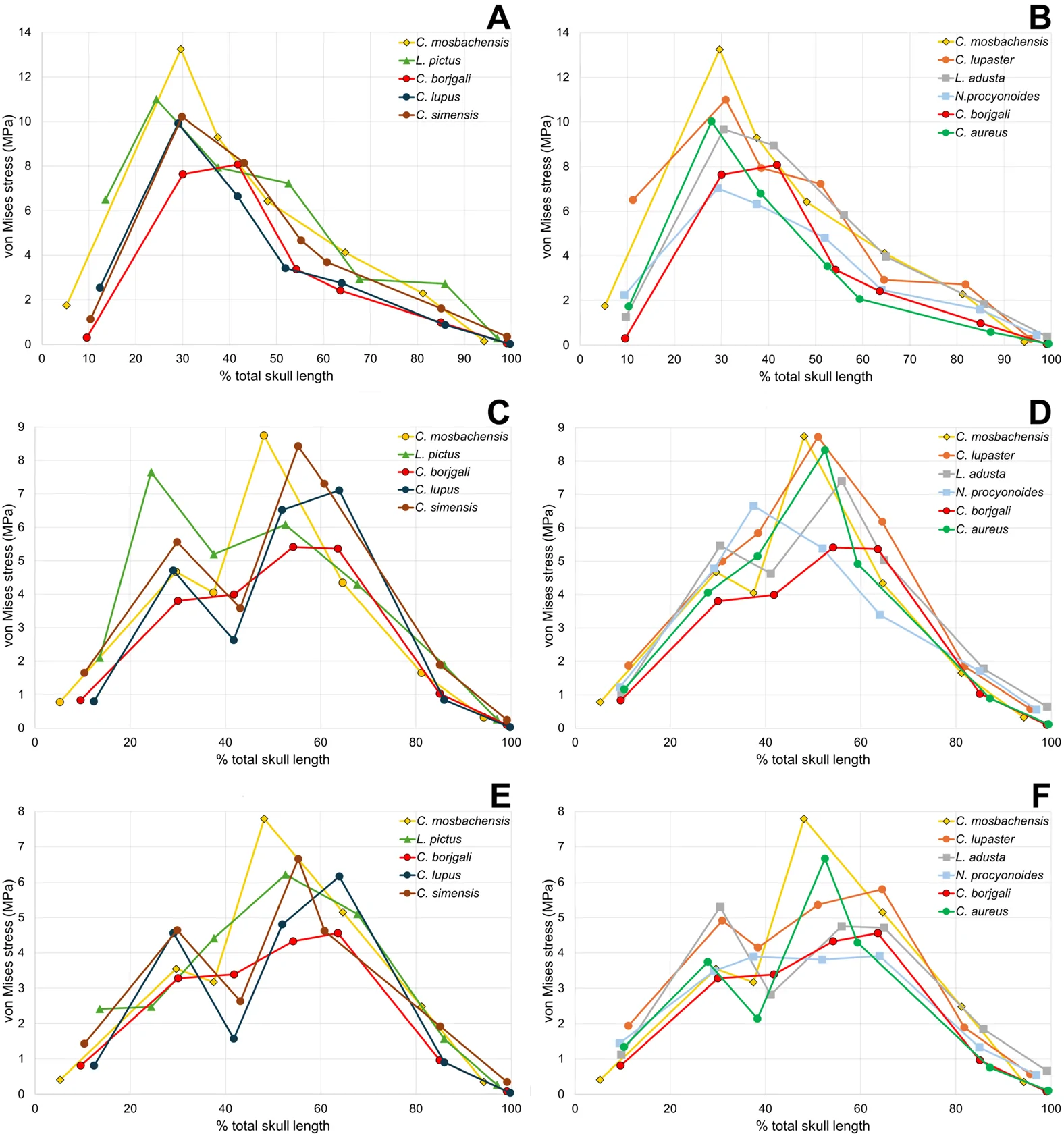
von Mises stress values (in MPa) of *C. borjgali, C. mosbachensis* and extant canids sampled along a sagittal plane passing through the right nasal and frontal bone and plotted on a graph in the three bite scenarios. Results are shown for (A, B) canine, (C, D) carnassial and (E, F) molar bite simulations. The extant species are divided into hypecarnivorous (left column) and meso-hypocarnivorous species (right column). The analogous anatomical sampling points (nasal, rostrum at infraorbital foramina, rostrum at anterior border of orbits, frontal region between postorbital processes, frontal region at the postorbital constriction, centre of the parietal bone and most posterior region of the dorsal cranium) are plotted as percentages of total skull length measured in dorsal view.

The canine bite simulation of *C. mosbachensis* exhibits broad stress patterns and relatively high von Mises stress values (figure 2G). An evident peak can be discerned at the base of the snout (values between 16 and 17.5 MPa), and it climbs toward the top of the frontals (values between 10.0 and 14.0 MPa). There are also two intense symmetrical peaks at the base of the zygomatics with particularly high values (even exceeding 30.0 MPa). In addition, relatively low stress values (light blue in figure 2G) affect broad portions of the rostrum and neurocranium, from the canines to the base of the zygomatic process of the temporal. Even the dorsal surface of the frontals displays such a uniform stress pattern, differing in this regard from *C. borjgali* (cf. figure 2F). In the bite simulations at the carnassial and molar of *C. mosbachensis* (figure 2G′,G″), a clear stress peak can again be observed at the base of the zygomatic with values from 17.5 up to 27.5 MPa. In the carnassial bite simulation, the orbital margin appears as a highly stressed area, in reason of the recorded values (17.0–25.0 MPa), that spreads with a diminishing trend towards the dorsal infraorbital foramen. Moreover, a broad and uniform pattern (5.0–12.5 MPa) covers a great portion of the cranium from the P3 dental alveolus to the zygomatic process of the temporal. As in the previous simulations, in the bite at the level of the molar tooth, extensive patterns covering large portions of the skull of *C. mosbachensis* can be observed. The orbital wall exhibits notably high stress values with a peak reaching about 24 MPa below the postorbital process. The particularly extensive patterns in all three load cases of *C*. *mosbachensis*, as well as the remarkably high von Mises stress values, suggest a cranium particularly subject to bite-related stress. This is supported even by the stress trend diagrams that show for *C. mosbachensis* the highest peaks among the tested species at the level of the infraorbital foramina (canine bite simulations) and between the postorbital processes (carnassial and molar bite simulations). Among the sample of extant species, *Canis lupaster* is the form which displays the closest stress distribution to that observed in *C. mosbachensis*, though in this latter, the values are usually higher (figure 2G–G″,D–D″). In the bite simulations at the canines and at the carnassial, both these species exhibit similarly expanded stress patterns at the base of the snout and above the P4, respectively (figure 2G,G′,D,D′). Moreover, the stress peak affecting the lateral wall of the orbit in the molar bite simulations assumes similar values and position in *C*. *mosbachensis* and *C*. *lupaster* (figure 2G″,D″). Some similarities can be discerned even with *Canis simensis* in relation to the presence and intensity of stress peaks on the orbital margin and at the base of the zygomatic in the load cases at the carnassial and molar teeth (figure 2G′,G″,C′,C″). Finally, both in the canine bite simulations of *C*. *mosbachensis* and *C*. *simensis,* there is no evident change in the stress values at the level of the frontal sinuses (figure 2C,G).

Observing the results among the extant species, some common features can be identified. In the simulations at the canines, a symmetric stress peak of varying extent and intensity affects the snout of all the tested species. The species in which such a peak is most extensive is *C. lupaster* (figure 2D), while in *Nyctereutes procyonoides,* it is barely visible (figure 2I). This evidence led us to consider the base of the snout as a bending point in the context of a bite simulation at the canines. *Lycaon pictus* represents a peculiarity in this regard: indeed, in this canid, the peak is not located in central position on the suture of the nasals, but the stress is laterally deflected towards the maxillae. As in *C*. *borjgali* and *C*. *mosbachensis*, in the extant species tested in this study, there is a lowering in the von Mises stress values in correspondence with the frontal sinuses as well as a loss of homogeneity in the pattern. Such a fall in the values varies in extent across the sample: it can be evident and wide in *C*. *aureus* and *C*. *lupus* (figure 2E,H), almost absent in *C*. *simensis* and *Lupulella adusta* (figure 2B,C) or in an intermediate condition like in *C*. *lupaster* (figure 2D). In *N*. *procyonoides,* the negative peak is evident but highly circumscribed to the rostralmost portion of the frontals. In the simulations of carnassial bite, the most evident common feature is the peak on the maxilla in correspondence with P4, as detected in *C*. *borjgali* and *C*. *mosbachensis* (figure 2F″, G″). Among the living species included here, such a high-stress area appears less pronounced in *C*. *aureus* (figure *E*′), while it is clearly noticeable in *C*. *simensis* and in *N*. *procyonoides* (figure 2C′,I′). Moreover, this pattern is particularly expanded, covering a wide portion of the maxilla, in *L*. *pictus*, *C*. *simensis* and *C*. *lupaster* (figure 2A′,C′,D′). Even in the molar bite simulations, there is an area where the tensile stress is concentrated, which corresponds to the lateral wall of the orbit. The bite simulations of *C*. *lupaster* and *C*. *simensis* exhibit a particularly expanded peak. In the latter, we also observe a high-stress area on the dorsal border of the zygomatic at the base of the orbit (figure 2C″). On the other hand, in *L. pictus,* the peak on the lateral wall of the orbit is poorly developed (figure 2A″).

In general, observing the von Mises stress patterns from the canine bite simulations and the relative stress trend graphs (figures 2A–I and 3A,B), *C. lupus* and *C. aureus* are the species that show the least stressed crania among the extant Canini tested here, while *C*. *lupaster* and *L*. *adusta* display the highest von Mises stress values. Even in the canine bite simulations of *N*. *procyonoides*, we record low stress values. Considering the results of the living sample in the simulations at the carnassial tooth (figures 2A′–I′ and 3C,D), we observe the most intense stress in *C*. *simensis* and in *C. lupaster*, while the least intense in *C*. *aureus*. Finally, coupling the observation of the stress patterns and the values sampled on the cranium in the bite simulations at the M1 (figures 2A″–I″ and 3E,F), *C. simensis* exhibits the highest stress values globally, while the lowest values are observed in *C. aureus* and *L. pictus*.

## 3. Discussion

### 3.1. Finite element analysis and ecology

Assessing the results from the modern sample, some interesting considerations can be addressed. Based on the stress patterns resulting from the canine bite simulations, the extension of the stress peak at the base of the snout seems to be somewhat correlated with the proportion of this anatomical district. Indeed, the broadest peak can be detected in *C. lupaster*, *C. simensis* and *L. adusta*, all having long and slender rostra. However, a precise correlation with the ecology is hard to establish. Remarkably, *C. lupaster* and *C. simensis*, despite the different ecologies (*C. lupaster* is a generalist mesocarnivore, while *C. simensis* is a specialist hypercarnivore), are known to feed on rodents [1,34]. In addition, *L. adusta* is a hypocarnivore that also includes small vertebrates and invertebrates in its diet [1,35]. Consequently, the elongation of the snout within the tribe Canini could be cautiously related to the feeding of small-sized and motile prey items, the capture of which does not generate high stress on the skull. The other hypocarnivore canid included in this sample is *N. procyonoides*, which displays a greatly shortened and robust rostrum, contrasting in this regard with *L*. *adusta*. Such a feature of the raccoon dog easily explains the good mechanical performance of its cranium in the bite simulations at the canines. *Nyctereutes procyonoides* is considered a true omnivore feeding upon an extremely wide range of food types (small mammals, birds, eggs, fish, amphibians, invertebrates and vegetal material) [35]. It is possible that such a broad diet does not require a specialization of the snout, thus explaining the morphologic difference with *L*. *adusta*. On the other hand, both *C*. *lupus* and *L. pictus* are known to be hypercarnivorous group hunters that usually catch large-sized vertebrates through social hunting [1,35]. This subjects the skull to great loads during hunting, owing to the catching of heavy and fighting prey. Nevertheless, *C*. *lupus* and *L*. *pictus* exhibit quite different cranial shape and structure: the former bears a moderately elongated rostrum and the latter a strongly shortened one. This is reflected by the deeply different stress patterns of these two species in their canine bite simulations. These discrepancies can be interpreted as different answers to the same pressure: *C*. *lupus* may limit the bending load at the base of the snout by proportions that give stiffness to the cranium and maybe with the aid of other structures, like the maxillary sinuses. On the other hand, *L*. *pictus* seems to deviate the stress on the wide lateral surface of the maxillae, possibly thanks to a more dorsally elevated shape of the snout compared with *C*. *lupus*. In addition, among the hypercarnivores, *L. pictus* exhibits low stress peaks on the lateral wall of the orbit and the orbital margin in the molar bite simulation, suggesting a cranial structure resistant to the solicitations related to mastication. This may be explained as an adaptation for the processing of harder parts of prey items (e.g. cartilage, tendons and possibly bones) in a highly competitive or temporarily poor environment. Remarkably, packs of African wild dogs were observed spending time consuming the skeletal portion of their kills when they are energetically stressed [36].

In the context of bite simulation at the carnassial tooth, the stress peak in correspondence with the P4 is particularly evident in *N*. *procyonoides*, which can be traced back to the morphology of the dorsal infraorbital foramen, anteriorly expanded into a deep groove on the maxilla. In addition to this, in the posterior bite simulations (i.e. carnassial and molar), the tensile stress accumulation on the maxilla and lateral wall of the orbit, respectively, is particularly broad and evident in *C. lupaster* and *C*. *simensis* (figure 2D′–D″,F′–F″), suggesting that their crania are not particularly adapted to deal with the load related to the mastication. This may be related to the consumption of small-sized prey items, such as rodents or hares, which do not offer high resistance. Across the three load cases, *C. simensis* is one of the species with the poorest mechanical performance owing to the extreme elongation of its cranium, highlighting and reflecting the specialized ecology of this species. However, a clear bond between ecology and biomechanics is hard to discern. As an example, *C. aureus* exhibits generally good mechanical performance, and *C. lupaster* exhibits far lower performance, but they are both considered mesocarnivores [1]. The way in which a cranium absorbs the load strictly depends on its morphology; however, different shapes and adaptations can respond to similar lifestyles and selective pressures (e.g. *C. lupus* and *L. pictus*). Moreover, the skull is a highly complex structure that fulfils diverse tasks (like sensory functions) in addition to biting and nutrition. Furthermore, even phylogeny has an influence on modelling cranial shape. Biomechanics is a powerful tool for exploring the ecology of vertebrate species, both living and fossil. However, to reconstruct the ecology (and palaeoecology) of vertebrates and understand how it influences the mechanics of the skull, it is necessary to couple digital simulations with other investigation techniques (e.g. geometric morphometric, microwear and isotopic analysis). Based on the von Mises stress data from *C. borjgali* (figures 1A–A″ and 3), we can affirm that this fossil canid bears a cranium well adapted to withstand the stress caused by a biting action when compared with the living species included in this study. Considering the values and their range of the von Mises stress patterns in the anterior bite simulations (i.e. simulations at the canines), the cranial structure of *C. borjgali* appears stiffer than those of *C. lupaster*, *C*. *simensis* and *L. adusta*. Possibly, this is bound to the major cranial elongation of these extant species relative to *C. borjgali*, as a longer lever arm accumulates more tensile stress. However, in the context of an anterior bite, the cranium of *C*. *borjgali* is more subjected to bite-related stress than *C*. *aureus* and *N. procyonoides*, owing to the higher intensity of the stress peaks at the base of the snout and orbital margin. This is probably related to the shortened rostrum observable in these species that gives major robustness to their crania compared with *C. borjgali*. Studying the results from the posterior bite simulations (i.e. simulations at the carnassial and first molar), the greater differences emerge in the comparison between *C. borjgali* and forms like *C. simensis* and *L. adusta*. Indeed, these forms seem structurally less adapted to withstanding the stress generated during a posterior biting action, and this is probably related to the elongated and lowered shape of the neurocranium. Among the species considered in this study, the cranium of *C. lupus* seems to have the most similar mechanical behaviour to that observed in *C. borjgali*, and thus similar resilience to bite-generated stresses. This is not completely unexpected considering the morphological and phylogenetic closeness between these two *Canis* species. Similar mechanical performance of the cranium may indicate similar mechanical demands and thus common adaptations to diet. Considering this, along with the morphological data, we tentatively hypothesize for *C. borjgali* similar dietary habits to those of the extant grey wolf, known to be a hypercarnivore feeding upon large-sized vertebrates [1]. The resulting von Mises stress pattern (figure 2A″,E″), as well as the sampled values on the dorsal surface (figure 3), show that the cranium of *C. borjgali* has slightly better mechanical performance than *C. lupus* in the molar bite simulation. Considering the function of the molar teeth and the simulated bite scenario, it is our opinion that this may be an adaptation for the consumption of hardened materials (e.g. grains, nuts and bones). Remarkably, Werdelin [28] described bone crushing as a widespread feeding behaviour among carnivorans, including *C. lupus*, consisting in the mastication of mineralized tissues using the wide basined molars [21,28,37]. Thus, the cranial stiffness observed in the specimens of *C. borjgali* examined here may be interpreted as an adaptation for the ingestion of harder parts of prey items, such as bones and cartilages. All things considered, we deem that the relatively stiff cranial structure of *C*. *borjgali* suggests feeding habits with non-negligible biomechanical demands, if compared with modern Canini.

On the other hand, the von Mises stress patterns and values suggest that the cranial structure of *C*. *mosbachensis* is more susceptible to the bending stresses generated during a biting action. This is particularly evident in the stress trend graphs (figure 3), in which *C*. *mosbachensis* displays the highest stress values at the base of the snout (canine bite) and on the frontal between the postorbital processes (carnassial and molar bites). This is probably related to its more gracile morphology with respect to *C*. *borjgali* and *C*. *lupus*, especially with respect to the thin and elongated snout. Among the living canids considered in this investigation, *C*. *lupaster* and *C*. *simensis* appear as the closest species to *C*. *mosbachensis* from a morphological point of view. This evidence is well supported by the results of the simulations, since the shape of the patterns as well as the peak values of the von Mises stress are highly comparable in *C*. *lupaster* and *C*. *mosbachensis* (figure 2B–B″,D–D″). Even the graphs representing the stress trends highlight quite similar mechanical behaviour for the crania of these two species, especially for the bite simulations at the canine and carnassial teeth (figure 3B,D). There are also a few resemblances in the results with *C*. *simensis*, like the stress values on the dorsal surface of the frontals (figure 2B–B″,F–F″) that could be traced back to the cranial elongation in *C*. *simensis*, somewhat recalling the proportions of *C*. *mosbachensis*. Since the greatest similarities of the mechanical reaction emerge in the comparison with *C*. *lupaster* of all the extant species, we can assume that the bite mechanics of *C*. *mosbachensis* were akin to that of a mesocarnivorous form. Indeed, *C*. *lupaster* includes in its diet rodents, plant material and insects [34,38,39]. Remarkably, both *C*. *mosbachensis* and *L*. *adusta* (the only hypocarnivorous Canini included here) exhibit a relatively elongated cranium. However, comparing the results of these two canids, the latter has generally lower stress values, especially at the carnassial and molar bite (figure 2B′–B″,G′–G″). This datum could be caused by a higher biomechanical demand of a hypocarnivorous diet, which also includes leathery non-meat food, with respect to the food habits of *C*. *mosbachensis*. Note that both *C*. *borjgali* and *C*. *mosbachensis* are representatives of the modern wolf clade, and they roamed the European area during the Early Pleistocene, but the bite simulations express highly different cranial mechanics in these two species, somewhat suggesting divergent dietary adaptation and possibly ecological role. Hence, these results emphasize the importance of the Early Pleistocene as a period of diversification for the *Canis* lineage [14], during which these carnivorans may have explored diverse ecological niches.

If we evaluate the von Mises stress patterns within the phylogenetic framework (figure 2), no clear phylogenetic signal seems to emerge from the present sample. Rather, the distribution of stress appears to vary according to both cranial architecture and the specific load case. In this context, the canine bite simulations display the most interesting results. Across these conditions, *L. pictus* displayed the most peculiar pattern relative to the sampled Canini, with the ventrolateral propagation of the von Mises stress at the base of the rostrum. Such a particularity is consistent with the phylogenetic position of *L. pictus*, which occupies the most external and basal position among the Canini considered here. This may reflect an alternative and distinctive morpho-functional adaptation with respect to the *Canis* and *Lupulella* lineages. However, without data from other species close to *L. pictus* from a morphological and phylogenetic perspective, it is not possible to state this with certainty. Within the *Canis* genus, the stress distribution maps highlight broadly comparable topographic patterns, with some recurrent stress concentrations in homologous cranial regions (e.g. the infraorbital region) and differences mainly related to the intensity and extension of the von Mises stress patterns. The carnassial and molar bite simulations show an even higher level of uniformity in the spatial distribution of the stress across the sampled taxa, with no clear separation among the main phylogenetic lineages. Again, major differences are mostly driven by the magnitude of the stress peaks in specific species. Within the clade of *C. borjgali*, *C. mosbachensis* and *C. lupus*, *C. mosbachensis* shows a cranium that is more susceptible to bite-related stress. This suggests that closely related taxa may still differ in cranial mechanical response, probably owing to species-specific differences in cranial architecture. At the same time, the lack of a clear correspondence between biomechanical results and phylogenetic relationships possibly indicates that the observed differences in von Mises stress distribution are more related to ecological and functional variation than to phylogeny alone. However, the limited taxonomic sample prevents us from a sure separation of the ecological signal effects from the phylogenetic history. Broader comparative analyses will be needed to shed light on the relative influence of ecology and phylogeny in the mechanical behaviour of the cranium across canids.

### 3.2. Frontal sinuses and biomechanics

Frontal sinuses are a pair of grossly symmetrical cavities located within the frontal bones, and they are part of the paranasal sinuses. Across Carnivora, several hypotheses were proposed to explain the presence and development of these anatomical structures, such as thermoregulation of the brain, improvement of sensory abilities, augmented resistance to muscular loads or support for hibernation [29,30,31,40,41]. In Canidae, the taxonomical value of frontal sinuses is widely recognized [6,32,42], as well as their relationship with diet and nutrition [26,42–44]. Recently, it has been hypothesized that these structures have a role in dealing with bite-generated cranial stress. The same authors suggest that the frontal sinuses play an important role in stress dissipation, especially during a biting action taken with the canines [27]. According to the interpretation of this study, the influence of the frontal sinuses on the biomechanics of the bite in canids seems positively correlated with the dorsal convexity of these cavities and the presence of bony struts, acting as reinforcements [27].

Within our sample, we can identify *C. borjgali*, *C. lupus* and *L. pictus* as species with particularly expanded frontal sinuses and adorned with bony struts (figure 4B,D,H) [32,45]. The other extreme is represented by *N. procyonoides* (figure 4I), which exhibits smooth frontal sinuses with a scarce development in volume and vertical length [32,45]. All the other species considered here display intermediate development conditions that are difficult to categorize rigidly. Studying the results of the anterior bite simulations performed here, in several species (*C. aureus*, *C*. *borjgali*, C. *lupaster*, *C*. *lupus*, *L*. *pictus* and *N*. *procyonoides*), we observe a fall in the stress values in correspondence with the area above the frontal sinuses (figure 2A,C–E,G,I). Among these, such an effect is more evident in *C*. *aureus*, *C*. *borjgali* and *C*. *lupus*, in which the stress pattern acquires an uneven appearance. Interestingly, in these species, the frontal sinuses are well developed and display a dorsally arched shape, particularly in *C*. *borjgali* and *C*. *lupus*. From a biomechanical point of view, the domed morphology of the frontal sinuses probably acts as a discharging structure to reduce the bite-related stress on the frontals [27]. Moreover, within the living sample, *C. lupus* bears the most arched and ornamented frontal sinuses (figure 4D) that are probably useful in improving the cranial stiffness to catch the large-sized mammals constituting the diet of this canid. In this regard, *L*. *pictus* represents a special case again, since its frontal sinuses are not particularly domed (figure 4H) and display a deep anterior concavity, followed almost perfectly by the stress patterns. In this species, the action of the frontal sinuses appears to be limited to the posteriormost region of the frontals. We deem that this condition is related to the peculiar cranial morphology of *L. pictus*, characterized by a shortened rostrum that contributes to laterally deviating the stress generated by an anterior bite. In this context, the frontal sinuses probably lack part of their biomechanical value, and thus they do not adopt the domed shape observed in *C. lupus*. In addition to these forms with enlarged, complex and dome-shaped frontal sinuses, our sample includes *N*. *procyonoides*, in which the frontal sinuses are small and dorsoventrally low vesicles located within the frontal bone (figure 4I). Interestingly, the influence of the frontal sinuses on the cranial reaction to an anterior bite load seems to be limited to a highly localized fall of the von Mises stress observable at the level of the postorbital processes. In our view, the poor development of these cavities explains the very limited effect on the frontal bones in the anterior bite simulation of *N. procyonoides*. Such a reduced influence is even more evident if we compare *N. procyonoides* with *C. borjgali* and *C. lupus*, in which the reduction of the von Mises stress values at the level of the frontals is more evident and extensive, probably owing to the greater development and domed morphology of the frontal sinuses of these two species. The ecological function of the small frontal sinuses in *N. procyonoides* is hard to understand; we cannot exclude that the presence of frontal sinuses in *N. procyonoides* may be a symplesiomorphic condition inherited from ancestors in which these structures were well developed (e.g. *Nyctereutes sinensis*, *N. tingi* and *N. vulpinus*) [46,47]. Note that *N*. *procyonoides* is part of the tribe Vulpini, characterized by the absence of frontal sinuses in its members [42]. Thus, the genus *Nyctereutes* evolved these structures independently from the tribe Canini. The evolutionary pressures that drove the appearance of frontal sinuses in *Nyctereutes* are still subject to debate, and they are beyond the aim of this paper. Nonetheless, a multi-disciplinary investigation focused on this canid genus, also including the fossil forms, will hopefully clarify the matter. The ecological significance of moderately domed frontal sinuses in *C. aureus* (figure 4A) is not an easy matter. Indeed, this species displays quite good mechanical performance in the anterior bite simulation, probably also owing to a shortened snout. The golden jackal (*C. aureus*) is known as a highly opportunistic forager [35,48]; in this context, a moderately robust cranial structure may be useful for its great versatility in diverse ecological contexts. Notably, some subspecies of *C. aureus* are known to include small ungulates in their diet, the hunting of which could cause significant stress on the skull [35]. Interestingly, *C. lupaster* displays frontal sinuses highly similar to *C. aureus*, with a moderate dorsal curvature and poorly developed bony struts. Despite this, in *C. lupaster*, the fall of the stress values is almost limited to the anterior portion of the frontals. This may be linked to the slightly more elongated snout of *C. lupaster*, with respect to *C. aureus*, which makes the action of the frontal sinuses somewhat less effective, worsening the mechanical performance of its cranium. On the other hand, the von Mises stress patterns of the anterior bite simulations in *C. simensis* and *L. adusta* suggest that in these species the frontal sinuses have a very limited or absent effect. Remarkably, these species exhibit a peculiar morphology of the frontal sinuses with a flattened dorsal surface (figure 4F,G), as well as the absence of developed bony struts. Furthermore, these species exhibit elongated and slender snouts that are probably functional for the capture of small prey items included in their diet [34,35]. In our view, such a feeding ecology does not cause high mechanical demands on the cranium; thus, in these species, there is no need for inflated frontal sinuses as in *C. lupus*. Remarkably, we observed similar stress patterns affecting the frontals of *C. mosbachensis*, along with non-inflated frontal sinuses (figure 4E) and a slender rostrum. Such a condition recalls what was observed in *C. simensis* and *L. adusta*, and this lead us to hypothesize that the palaeoecology of *C*. *mosbachensis* had low mechanical demands, like in these two modern canids. In canids, the frontal sinuses seem to have a biomechanical value during a biting action performed at the level of the anteriormost teeth. These cavities act by reducing the bite-generated load on the cranium, and this effect is more relevant as the frontal sinuses are dome-shaped. These results prove that even the cranial proportion represents an important parameter, as a slender rostrum is more vulnerable to the stress generated by a biting action.

**Figure 4.**
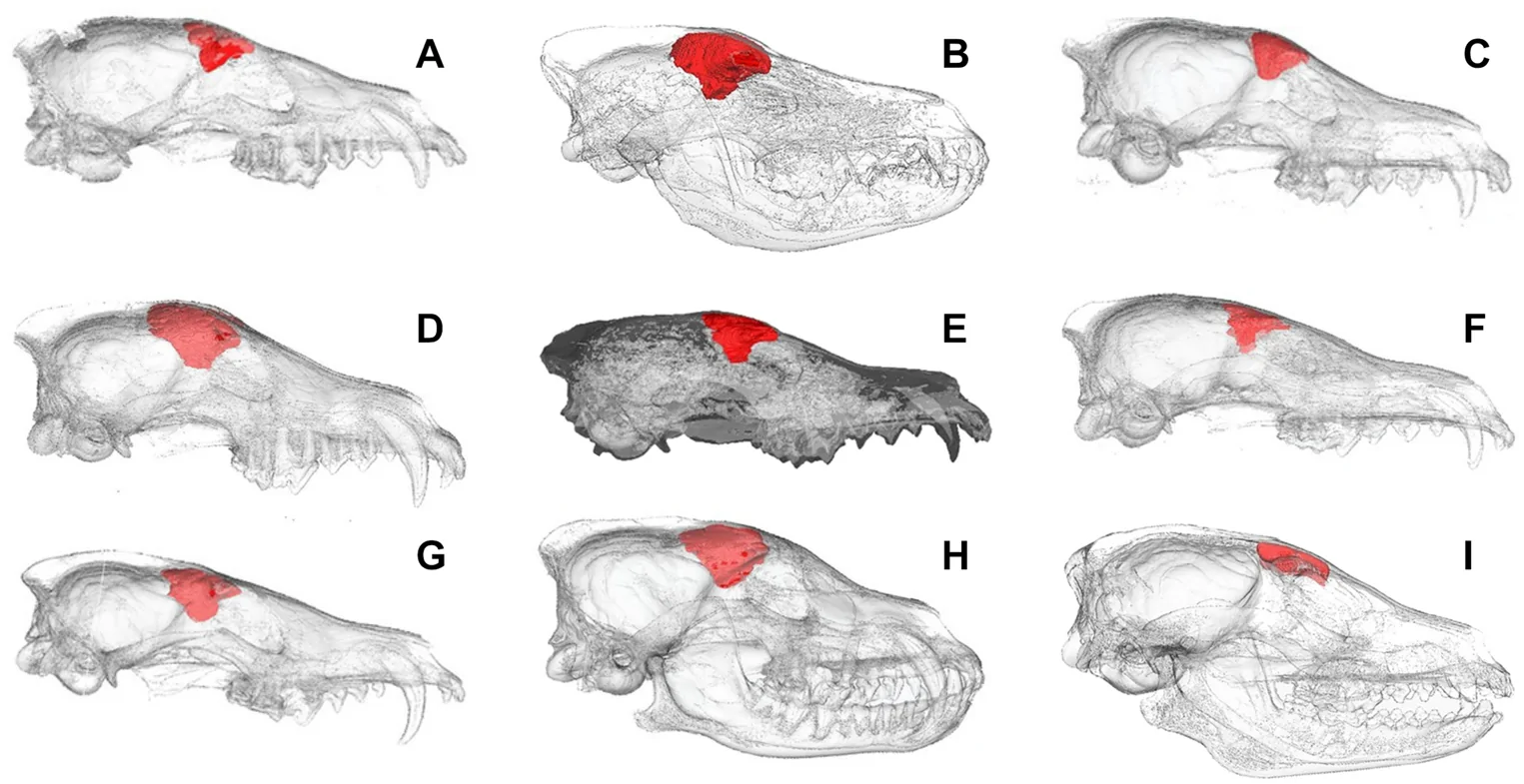
Crania of the fossil and extant species of Canidae included in this study with the frontal sinuses in natural position. (A) *C. aureus*, (B) *C. borjgali*, (C) *C. lupaster*, (D) *C. lupus*, (E) *C. mosbachensis*, (F) *C. simensis*, (G) *L. adusta*, (H) *L. pictus* and (I) *N. procyonoides*. The crania are not to scale. Modified after Frosali *et al.* [32] and Frosali *et al.* [45].

Observing the von Mises stress patterns in the posterior bite simulations (figure 2A′–I′,A″–I″), the influence of the frontal sinuses is not obvious within the tested sample. In *C. lupus* and *C. borjgali*, there are localized areas with low-stress values on the dorsal surface of the frontal that are particularly evident in the bite simulations at the M1 and having a grossly rostrocaudally elongated shape. These patterns may be related to the presence of developed bony struts ornamenting the internal walls of the frontals, jutting within the sinuses of these species, that consequently appear to act as supporting or reinforcing structures against bending, as previously suggested by former studies [49,50]. In addition to this, in the posterior bite simulations of *L. pictus*, the stress patterns on the frontal show local falls of the stress values in the vicinity of the postorbital process (figure 2H′,H″). These variations are discernible even in the stress trend diagrams, in which the line representing *L. pictus* does not show the peaks detected in the other hypercarnivorous species. However, it is hard to understand if the cranial strength experimentally observed in the posterior bite simulations of *L. pictus* was due to the frontal sinuses or to the shortened and robust morphology of its cranium. Notably, the frontal sinuses of *L. pictus* do not exhibit the deep bony struts observed in *C. lupus* and *C. borjgali*. However, there could be other factors, like the total volume or the vertical development, that influence the biomechanical action of the frontal sinuses, that should be investigated in future analyses. In our view, it is highly plausible that both cranial proportions and the morphology of the frontal sinuses influence the biomechanics of the cranium. In support of this, observing the von Mises stress values sampled on the neurocranium of *N. procyonoides* in the posterior bite simulations, such a species shows a remarkable cranial strength in these load cases. From the ecological point of view, this can be considered functional for the processing of hard non-meat material that constitutes an important portion of its diet [35,48]. However, the mechanical reason behind these results can be traced back to the short and robust structure of the cranium observed in *N*. *procyonoides*, since its frontal sinuses are extremely reduced.

Within our sample, the frontal sinuses seem to have an important relieving action on the stress generated by an anteriorly located bite. In general, the magnitude of this effect appears to be positively related to the dorsal convexity of these cavities. Our data also suggest that the presence of well-developed bony struts is an element that helps in dealing with the bite-related load, especially in the posterior bites. Remarkably, the results of this study are in good agreement with those of the study focused on *E. davisi* mentioned above [27], and they improve our knowledge on the biomechanical significance of the frontal sinuses in relation to the biting action. Possibly, future investigations focused on the integration of the digital simulations with other parameters will refine our knowledge on the link between frontal sinuses and mechanical behaviour of the cranium.

## 4. Conclusions

In this study, FEA was used to simulate the bite of the fossil canids *C. borjgali* and *C. mosbachensis* to estimate the reaction stress of their crania in three different bite scenarios. The resulting stress patterns were compared with those of a broad selection of extant canids, having a wide spectrum of ecologies. The aim of this work was to obtain new insights into the feeding capabilities of *C. borjgali* and *C. mosbachensis* to better understand the evolutionary dynamics of the modern wolf lineage. In addition to this, the data from the bite simulations were employed to evaluate the influence of the frontal sinuses on the mechanics of the cranium during the biting action. The von Mises stress data show good mechanical performance for the cranium of *C. borjgali* close to those of *C. lupus*. Probably, *C. borjgali* was able to process even the hard parts of its food items, recalling the bone crushing observed in the extant grey wolf. Unlike *C. borjgali*, *C. mosbachensis* displays a relatively weak cranial structure with high stress values, suggesting that its diet did not include hard food difficult to process. Remarkably, the extant species among those considered here with the most similar mechanical behaviour is *C. lupaster*, a canid known to feed upon several small vertebrates (e.g. rodents and reptiles). The results presented from the FEA simulations suggest that the frontal sinuses contribute to the dissipation of the bite-related stress, especially in the context of an anterior bite. A fundamental factor seems to be the presence and degree of dorsal convexity of the frontal sinuses that gives these cavities a dome-shaped morphology; even the presence of developed bony struts that behave as reinforcement structures, whose effect is more evident in the posterior bite simulations.

The present study highlights new aspects of the feeding capabilities and possible ecologies of *C. borjgali* and *C. mosbachensis*, two important representatives of the *Canis* genus. Interestingly, these two species are chronostratigraphically and possibly phylogenetically close; nevertheless, these two canids display different bite mechanics and thus probably dissimilar palaeoecology. This difference emphasizes the complexity of the evolutionary framework of the genus *Canis* as well as the need for additional studies. Biomechanical analyses that include fossil representatives of extant lineages, such as this one presented here, are extremely useful tools to unravel the evolutive dynamics and the selective pressure that guided the evolution of the clade of modern wolves and jackals.

## Supplementary Material



## Data Availability

Data are provided in the supplementary material [51].
