# Supplementary material for: Bite mechanics within the wolf lineage: feeding capabilities of Canis borjgali and Canis mosbachensis inferred by digital bite simulation
Source: R Soc Open Sci. 2026 Oct 7;13(10):261396. doi: 10.1098/rsos.261396 (PMC13641898; doi:10.1098/rsos.261396)
Supplement: Supplementary file 1 [file rsos.261396.zip › rsos.261396/rsos261396_si_001.pdf]

Bite mechanics within the wolf lineage: feeding capabilities of *Canis borjgali* and C. *mosbachensis* inferred by digital bite simulation

Emanuele Peri[1], Alberto Corridori[1], Saverio Bartolini-Lucenti[1],

David Lordkipanidze[2*], Lorenzo Rook[1*]

[1]*Department of Earth Science, Paleo[Fab]Lab, University of Florence, Florence, Italy*

[2]*Georgian National Museum, Tbilisi 0105, Georgia; Department of Natural History, Tbilisi State University, Tbilisi 0179, Georgia*

**Corresponding authors – dlordkipanidze@museum.ge; lorenzo.rook@unifi.it*

Orcid:

Emanuele Peri 0000-0001-8635-5379

Alberto Corridori 0009-0009-5458-7656

Saverio Bartolini-Lucenti 0000-0003-1280-5378

David Lordkipanidze 0000-0003-4894-4038

Lorenzo Rook 0000-0001-8923-5428

Materials and methods

Institutional abbreviations

BLOC Bartolini-Lucenti Osteological Collection, private collection, Florence (Italy);

CBL Collezione Borzatti-Lowenstein, Museum of Anthropology and Ethnology, University of Florence, Florence (Italy);

CE Giacomo Doria Natural History Museum, Genoa (Italy);

IMEDEA Mediterranean Institute for Advanced Studies, Mallorca (Spain);

IVPP Institute of Vertebrate Paleontology and Paleoanthropology, Chinese Academy of Sciences, Beijing (China);

MG-GNM S. Janashia Museum, National Museum of Georgia, Tbilisi (Georgia);

MZUF La Specola Zoological Museum, University of Florence, Florence (Italy);

USNM Smithsonian National Museum of Natural History, Washington (USA);

Sample availability

This study focuses on crania referred to *Canis borjgali* (MG-GNM-D64) and *Canis mosbachensis* (IMEDEA-C2) from the Early Pleistocene from Dmanisi (1.8 Ma) and Cueva Victoria (0.9 Ma), respectively.

The comparative sample for our analyses included the following specimens as modern analogues of comparison:

Canis aureus (CBL-298)

C. lupaster (MZUF-1851)

C. lupus (MZUF-11874)

C. simensis (CE-818)

Lupulella adusta (MZUF-8496)

Lycaon pictus (USNM-368441)

Nyctereutes procyonoides (BLOC-20723)

We included the species of Canini listed above to cover the wide range of feeding ecologies displayed by this clade (hypercarnivore group hunter, hypercarnivore of small prey, mesocarnivore and hypocarnivore). In addition, such a sample is well representative of the morphological variability displayed by extant Canini, including forms with a short and robust snout (*L. pictus*), species having a more elongated ad slender skull (e.g., *C. simensis*) as well as intermediate morphologies (e.g., *C. aureus*). Besides these species, we also considered *N. procyonoides* to observe the stress patterns in a representative of the tribe Vulpini bearing frontal sinuses, which usually are lacking in this tribe.

The CT-scan of the specimen of *C. borjgali* considered here was performed at the Oncology Centre and Medical-Diagnostic Centre of the University of Tbilisi (Tbilisi, Georgia) and the scan of the *C. mosbachensis* specimen at Mútua Terrassa University Hospital (Terrassa, Spain).

Analytical methods

All the specimens were examined by medical CT-scan, and the image stacks were imported within the open-source software 3D Slicer 5.6.1 (https://www.slicer.org/) [1]. A first rough segmentation of the crania was obtained using threshold-based algorithms, which select the voxels within a density range. Subsequently, the segmentations were refined manually. Since we were interested in testing the mechanical behaviour of the cranium during a biting action, we did not include in the segmentations the turbinates and most of the cribriform plate. Indeed, these bones are thin and covered of soft tissue, consequently they should not greatly affect our mechanical results [2]. Moreover, to lighten our simulations and considering that this study is focused on the cranium, we filled the cavities of the mandibles obtaining a so-called cavity-filling 3D mesh. Finally, the segmentations representing crania, dentitions and mandible were exported from 3D Slicer as separate .STL files. All the exported 3D models were remeshed using the remesh algorithm of Auodesk Meshmixer; this passage was aimed to improve their topology. The 3D meshes were imported within the open-source software Blender 4.2.1 (https://www.blender.org/) and the crania and mandibles were aligned setting a jaw gape angle of 45°. For *C. borjgali* and *C. mosbachensis*, the damaged parts (e.g., postorbital processes and zygomatic arches) were reconstructed using the preserved portions of the specimens as reference (mirroring) as well as the morphology of strictly related canid taxa (e.g., *C. lupus*). Furthermore, since IMEDEA-C2 does not include any mandibular remain, we used a 3D scan of IVPP LOC13-74-166 including a left mandibular ramus referred to the Chinese species *C. variabilis* to build a mandible for the alignment with the *C. mosbachensis* specimen used in this study. Notably, *C. variabilis* is strictly morphologically close to the European form *C. mosbachensis* to the extent that some scholars consider the former as a

subspecies of the latter [3]. Considering this, we assumed IVPP LOC13-74-166 as a good choice for the purposes of our bite simulations.

Biomechanical simulations on taxa having different size may be influenced by scaling effects, and consequently led to biased conclusions [4,5]. To avoid this, we maintained the same input force/surface area ratio in all the simulations, scaling all the crania to the surface area of *C. lupus* (MZUF-11874) (109717 mm^2) and using the muscle force estimated for this specimen. Notably, since we employed a linear Finite Element (FE) solver, any constant input force/surface area ratio could be applied [4]. The force exerted by the masticatory muscles of *C. lupus* was estimated using the dry-skull method, thus multiplying the anatomical cross-section area of the muscle by the specific muscular tension [6]. For our purposes, the specific muscular tension was considered equal to 30 N/cm^2, following what previously did in many other papers investigating the mammalian bite [6,7,8,9,10]. This value has been recognised as slightly underestimated if referred to the mandible elevator muscle of mammals due to their pennation and variation of the fibre length [7,11,12,13]. However, we opted for this solution as considered a conservative estimation ensuring repeatable results [7]. The final value of total input force produced by the mandible elevator muscle of MZUF-11874 was estimated at 2343 N as specified below.

Data used for the FEA simulations

In order to maintain a constant input force/surface area ratio, we used in all the simulations the data from the *Canis lupus* specimen used in this study (MZUF-11874)

**Cranial surface area**	109717 mm^2
**Cross-section area of the temporalis muscle**	1825 mm^2
**Cross-section area of the masseter and pterygoid muscles**	2080 mm^2
**Specific muscular tension**	30 N/cm^2
**Input force of the temporalis muscle**	547.5 N
**Input force of the masseter and pterygoid muscles**	624 N
**N° of trusses of the temporalis muscle**	65
**N° of trusses of the masseter muscle**	27
**N° of trusses of the pterygoid muscle**	12

**Elastic module of the bone**	13.7 GPa
**Elastic module of the teeth**	38.6 GPa
**Poisson's ration of the bone**	0.3
**Poisson's ration of the teeth**	0.4

Finite element model

The meshes of the crania, teeth and mandibles were exported as .STL files and loaded in Strand7 R3.1.5 (https://www.strand7.com, Strand7 Pty. Ltd., Sydney, Australia) for the creations of the solid mesh and the setting of the boundary conditions. After a cleaning of the 3D models, we used the tool "automesh from plates" to generate solid meshes made of 4-noded tetrahedral elements (Fig. 1). The number of tetrahedral elements varied between 2,638,957 and 3,657,249 for the cranium, and between 523,174 and 3,189,790 for the dentition. Following previously published procedures, we assigned to the crania and teeth values of elastic modulus and Poisson's ration that are typical for mammalian bone ($E = 13.7$ GPa, $\upsilon = 0.3$) and dentine ($E = 38.6$ GPa, $\upsilon = 0.4$) [4, 7, 8, 9]. To simulate the temporo-mandibular joint, we placed two fixed constraints at level of the mandibular fossae allowing only the rotation around the axis perpendicular to the sagittal plane, following a procedure previously employed [2]. Similarly, we placed two constrains on the mandible at the level of the mandibular condyles roughly in correspondence of those placed on the cranium. To bound the teeth to the cranium, we used a series of links both at the crown-root interface and the root apices (5 links on single-rooted teeth, 8 links on double-rooted teeth and 9 on triple-rooted teeth). These entities have the function of transmitting the displacement and/or rotation from one node to another. For the simulations performed here, the following muscular groups were considered for the elevation of the mandible: the temporalis group, the masseter group and the pterygoid group. For the origins and attachment areas of the masticatory muscles, we based our work on the published material concerning the domestic dog [14, 15] and on the careful analysis of the morphology of the examined samples. Considering the taxonomic and morphological closeness of the fossil species examined here with the extant taxa, this approach should provide a realiable reconstruction of the

muscular architecture, even for the fossil taxa. The muscles were simulated in Strand7 using pretensioned beam elements transmitting an axial compression, defined as trusses (Fig. 1). The tension was calculated dividing the muscle force estimated with the dry-skull method by the number of trusses assigned to the relative muscle group. The number of trusses for each cranial side was 104: 65 for the temporalis muscle, 27 for the masseter muscle and 12 for the pterygoid group. As did in other published papers [2,7,8,9,12,13], the contact with the food item was simulated through a fixed constraint placed on the tip of the teeth involved in the simulations. To expand the bite scenarios examined in this study, we performed diverse simulations varying the position of the constraints on teeth. Consequently, we simulated:

- bilateral canine bite, two constraints at the tips of the C;
- unilateral carnassial bite, one constraint at the paracone of the P4;
- unilateral molar bite, one constraint at the protocone of the M1.

For the postprocessing of the results, the cranial reaction to the solicitations was expressed as von Mises stress, a widely accepted combined measure of the major stresses affecting a body under ductile failure condition and particularly valid for cortical bones [2,5]. The mechanical efficiency of the tested crania was assessed and discussed through the study of the von Mises stress values and distribution. We also assessed the biomechanical results even in light of the phylogenetic framework of Canidae in order to verify the possible presence of a signal from the evolutive history of this family. Furthermore, we sampled the values of the von Mises stress on the dorsal surface of the cranium at specific points (nasal, rostrum at infraorbital foramina, rostrum at anterior border of orbits, frontal region between postorbital processes, frontal region at the postorbital constriction, centre of the parietal bone, and posterior region of the dorsal cranium) along a plane passing through the right nasal and frontal bone and plotted on a graph, following literature [2]. Stress values in the vicinities of constrains or link elements were not considered in the biological interpretation, since likely affected by artificially high stresses. The comparison between the von Mises stress patterns observed in *C. borjgali* and *C. mosbachensis* and those of the extant species

allowed us to speculate about the paleoecology of the fossil forms in relation to their morphological adaptations.

To evaluate possible influences of the volumetric distribution of the brick elements on unweighted statistical descriptors of the von Mises stress distribution we calculated the Percentage Error of the Arithmetic Mean (PEofAM) and Percentage Error of the Median (PEofM) for each cranial model [16]. We limited our calculations to the cranium, as this was the target bone district of this investigation. Because the same cranial meshes and input force magnitude were used across the three simulated load cases, PEofAM and PEofM were calculated for the canine bite simulation and used as a diagnostic reference.

The resulting values of PEofAM and PEofM are reported in table 1. PEofAM values ranged from 4.61% to 14.55%, exceeding the 2% threshold proposed by Marcé-Nogué et al. (16). PEofM values ranged from 0.38% to 9.99%, exceeding the 5% threshold in four of the nine sampled species. These results suggest a certain distance of the models generated in Strand7 from ideally uniform mesh, particularly for the arithmetic mean. However, this does not invalidate the main analyses of the present study since interspecific comparisons have been based on the spatial distribution and local values of von Mises stress in comparable bite simulations, rather than on unweighted arithmetic mean or median. Moreover, all models have been generated using the same meshing protocol and boundary conditions, and all cranial meshes include several million elements, indicating a high level of spatial detail. Therefore, the observed differences in mechanical performance are unlikely to be driven primarily by differences in mesh element number or mesh heterogeneity, and are more plausibly interpreted as reflecting structural differences among crania.

1 Fedorov, A., R. Beichel, J. Kalpathy-Cramer, J. Finet, J.-C. Fillion-Robin, S. Pujol, C. Bauer, D. Jennings, F. Fennessy, M. Sonka, J. Buatti, S. Aylward, J. V. Miller, S. Pieper, and R. Kikinis. 2012. 3D Slicer as an image computing platform for the Quantitative Imaging Network. Magnetic Resonance Imaging 30:1323–1341.

2 Tseng, Z. J., and X. Wang. 2010. Cranial functional morphology of fossil dogs and adaptation for durophagy in *Borophagus* and *Epicyon* (Carnivora, Mammalia). Journal of Morphology 271:1386–1398.

3 Jiangzuo, Q., J. Liu, J. Wagner, W. Dong, and J. Chen. 2018. Taxonomical revision of fossil *Canis* in Middle Pleistocene sites of Zhoukoudian, Beijing, China and a review of fossil records of *Canis mosbachensis variabilis* in China. Quaternary International:1–16.

4 Slater, G. J., E. R. Dumont, and B. Van Valkenburgh. 2009. Implications of predatory specialization for cranial form and function in canids. Journal of Zoology 278:181–188.

5 Dumont, E. R., I. R. Grosse, and G. J. Slater. 2009. Requirements for comparing the performance of finite element models of biological structures. Journal of Theoretical Biology 256:96–103.

6 Thomason, J. J. 1991. Cranial strength in relation to estimated biting forces in some mammals. Canadian Journal of Zoology 69:2326–2333.

7 Figueirido, B., S. Lautenschlager, A. Pérez-Ramos, and B. V. Valkenburgh. 2018. Distinct predatory behaviors in Scimitar- and Dirk-Toothed Sabertooth Cats. Current Biology 28:3260-3266.e3.

8 Figueirido, B., S. Tucker, and S. Lautenschlager. 2024. Comparing cranial biomechanics between *Barbourofelis fricki* and *Smilodon fatalis*: Is there a universal killing-bite among saber-toothed predators? The Anatomical Record n/a.

9 Janis, C. M., B. Figueirido, L. DeSantis, and S. Lautenschlager. 2020: An eye for a tooth: *Thylacosmilus* was not a marsupial "saber-tooth predator". PeerJ 8:e9346.

10 Wroe, S., M. Colin, and T. Jeffrey. 2005. Bite club: comparative bite force in big biting mammals and the prediction of predatory behaviour in fossil taxa. Proceedings of The Royal Society B: Biological Sciences 272:619–625.

11 McHenry, C. R., S. Wroe, P. D. Clausen, K. Moreno, and E. Cunningham. 2007. Supermodeled sabercat, predatory behavior in *Smilodon fatalis* revealed by high-resolution 3D computer simulation. Proceedings of the National Academy of Sciences 104:16010–16015.

12 Snively, E., J. M. Fahlke, and R. C. Welsh. 2015. Bone-Breaking Bite Force of *Basilosaurus isis* (Mammalia, Cetacea) from the Late Eocene of Egypt Estimated by Finite Element Analysis. PLOS ONE 10:e0118380.

13 Peri, E., P. L. Falkingham, A. Collareta, and G. Bianucci. 2022. Biting in the Miocene seas: estimation of the bite force of the macroraptorial sperm whale *Zygophyseter varolai* using finite element analysis. Historical Biology 34:1916–1927.

14 Hermanson, J. W. 2012. The Muscular System. Pp.185–280 *in* H. E. Evans, ed. Miller's Anatomy of the Dog. Elsevier Health Sciences.

15 Barone, R. 1980 Anatomia comparata dei mammiferi domestici. Vol. 1: Osteologia. Terza edizione italiana a cura di Ruggero Bortolami ed Emilio Callegari (2017). Edizioni Agricole New Business Media, Milano

16 Marcé-Nogué, J., S. de Esteban-Trivigno, C. Escrig, L. Gil. 2016. Accounting for differences in element size and homogeneity when comparing finite element models: armadillos as a case study. Palaeontologia Electronica 19.2.2T: 1–22.

Table S1

Species	N° elements	|PEofAM|	|PEofM|
Canis borjgali	3,575,588	6.62%	9.99%
Canis mosbachensis	2,693,380	14.55%	2.35%
Canis aureus	3,200,903	5.72%	7.86%
Canis lupaster	3,283,793	7.65%	7.35%
Canis lupus	3,062,925	6.96%	4.48%
Canis simensis	2,638,957	6.79%	9.01%
Lupulella adusta	3,657,249	4.61%	3.49%
Lycaon pictus	3,164,719	8.48%	0.38%
Nyctereutes procyonoides	3,306,454	7%	4.29%

Table S1 - Number of elements of the finite element mesh and mesh-homogeneity diagnostics for each analysed taxon. PEofAM (Percentage Error of the Arithmetic Mean) and PEofM (Percentage Error of the Median) were calculated following Marcé-Nogué et al. [16]. Element counts refer only to the cranium, which represents the anatomical region analysed in this study. PEofAM and PEofM values are reported as absolute percentages.

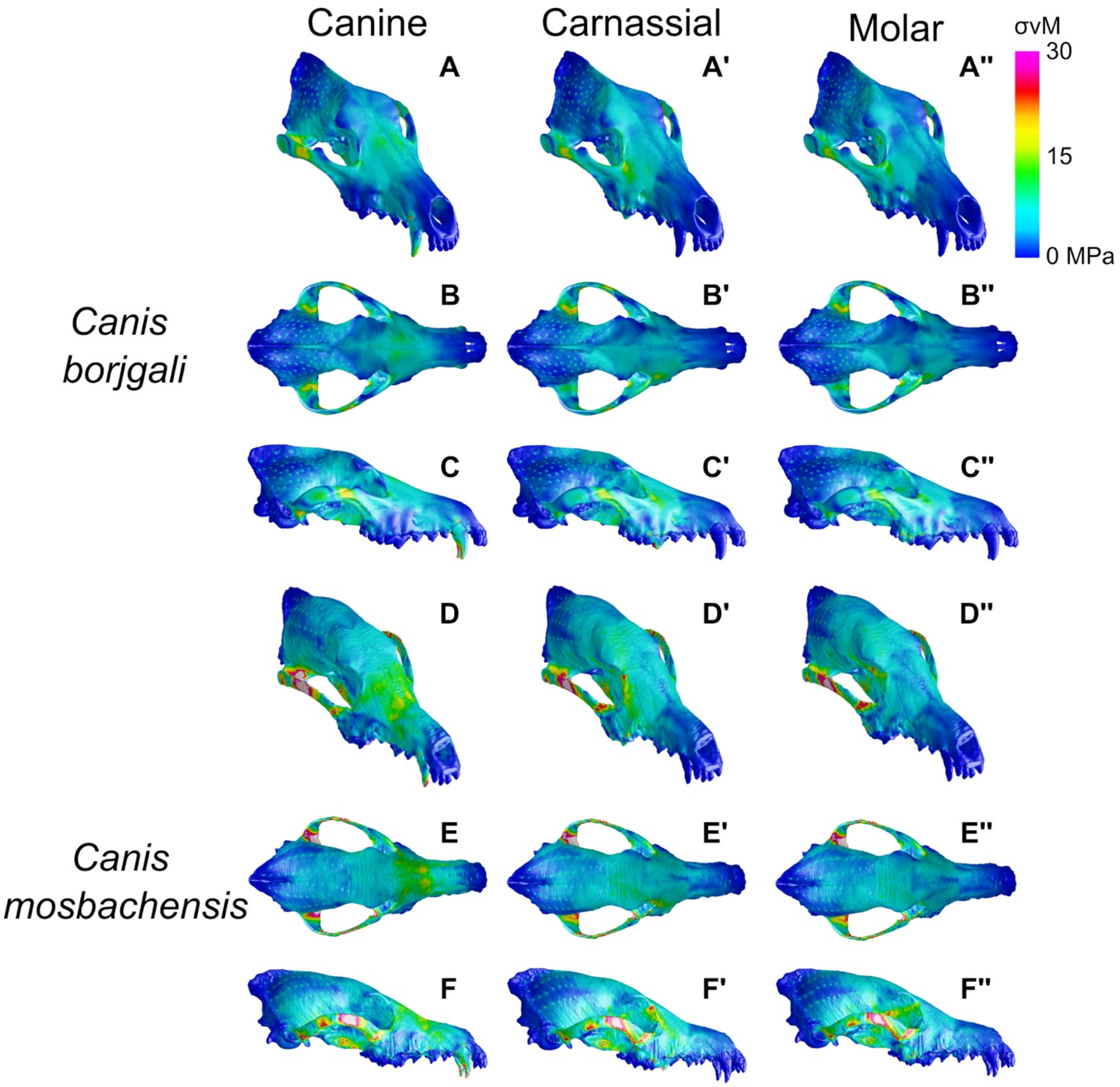

Figure S1 - Distribution maps of von Mises stress (σ_{vM}) resulting from the bite simulations at the canine teeth (**A**-F), carnassial (**A'**, **F'**) and M1 (**A"**, **F"**) on *Canis borjgali* and *C. mosbachensis*. The crania are in anterolateral view (**A-A"**; **D-D"**), cranial (**B-B"**; **E-E"**) and right lateral (**C-C"**; **F-F"**) views, and not to scale. Note that the dotted pattern on the neurocranium of are due to the punctiform force exerted by the trusses simulating the muscle fibres.
